# Right hepatectomy for primary monophasic synovial sarcoma of the liver: case report and review of the literature

**DOI:** 10.1016/j.ijscr.2025.111348

**Published:** 2025-04-23

**Authors:** Moctar Noufou Fodiya, Oumayma Lahnaoui, Mouna Khmou, Mohammed Anass Majbar, Amine Souadka, Amine Benkabbou

**Affiliations:** a Department of Digestive Oncology Surgery, National Oncology Institute Sidi Mohamed Ben Abdellah, Ibn Sina University Hospital Center, Mohamed V University, Rabat, Morocco; b Anatomopathology Department, National Oncology Institute Sidi Mohamed Ben Abdellah, Ibn Sina University Hospital Center, Mohamed V University, Rabat, Morocco; c Faculty of Health Sciences, Abdou Moumuni University, Niamey, Niger

**Keywords:** Synovial sarcoma, Liver, Monophasic, Case report, Immunohistochemistry, Right hepatectomy

## Abstract

**Introduction:**

Synovial sarcoma is a soft-tissue sarcoma whose histological origin has long been poorly understood. It occurs mainly near the extremities of larges joints but can occur anywhere in the body. Synovial sarcoma of hepatic origin is extremely rare, with only 13 cases reported in the literature.

**Case presentation:**

We report the case of a 68-year-old female patient who underwent right hepatectomy for a single 12.5 cm mass arising in the S6-S7 segments and evolving for over three years, long considered to be hepatocellular carcinoma. An Anatomopathological examination of the operative specimen showed an undifferentiated tumoral process. The immunohistochemical complement demonstrated a monophasic synovial sarcoma.

**Clinical discussion:**

Clinical and radiological signs are not specific, and diagnosis is based on histology, of which there are three types. Complete surgical excision remains the best therapeutic option for localized forms. It may or may not be combined with adjuvant chemotherapy in other forms, although the prognosis of the disease remains guarded.

**Conclusion:**

Synovial sarcoma of the liver is a rare disease with a histological diagnosis. Treatment is not standardized, and surgical excision remains the best option for localized forms.

## Highlights

• Primary synovial sarcoma of the liver is a rare disease. only 13 cases have been reported in the literature.
• We describe the case of a 68-year-old woman whose disease occupied almost the entire right lobe with a review of the literature.
• Symptoms are not specific, making histological diagnosis essential.
• Emphasizes the importance of R0 excision in the traitement and prognosis of the disease.

## 1 Introduction

Soft tissue sarcomas are malignant tumors that develop at the expense of "soft tissue." These include muscles, tendons, fat, blood and lymph vessels, nerves, and the tissues surrounding joints. As soft tissues are distributed throughout our body, soft tissue sarcomas are likely to occur in all parts of the body [1]. Primary synovial sarcoma of the liver is extremely rare. After a thorough literature search, only 13 cases of primary synovial sarcoma of the liver were reported [[2], [3], [4], [5], [6], [7], [8], [9], [10], [11], [12], [13]].

We present a very rare case of primary monophasic synovial sarcoma of the liver in a 68-year-old female with a chronic course whose radiological images suggested hepatocellular carcinoma, taken care of in our structure, which is a university institution. The work was reported in accordance with SCARE criteria [14].

## 2 Case presentation

**Fig. 1:**
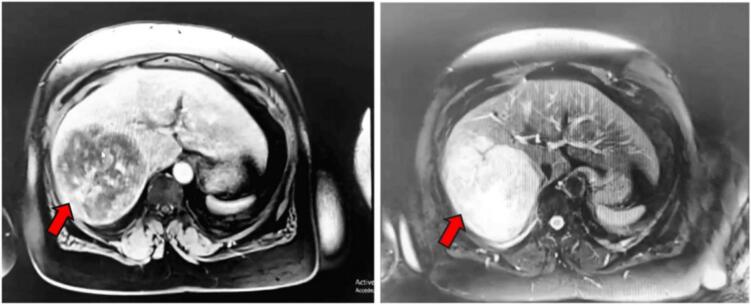
Hepatic MRI image showing the mass occupying segments 6 and 7 indicated by the red arrow. (For interpretation of the references to colour in this figure legend, the reader is referred to the web version of this article.)

This 68-year-old female patient is from South Saharan Africa; without any particular medical history, except obesity (Body mass index of 36.51), and a moderate alcohol consumption. Three years prior, she presented a constant abdominal pain located in the right hypochondrium. Initial imaging revealed a tumor located in segments 6 and 7 of the liver without underlying liver disease, suggestive of a fibrolamellar hepatocellular carcinoma. A first biopsy was performed but was inconclusive. She had refused surgery then and was placed under clinical surveillance. In July 2023, the patient accepted surgery and was referred to our center for surgical management. Clinical examination revealed pain in the right hypochondrium radiating to the right shoulder, with no nausea or vomiting, no jaundice and no pruritus. The laboratory work-up requested was normal, the most important are the following with the reference values in parentheses: Hemglobin 13.6 g/dl (Reference values: 11.3 to 16 g/dl); white blood cells 6000/μl (Reference values: 3800 to 11,000/μl); platelets 269,000/μl (Reference values: 15,000 to 45,000/μl); prothombin 100 % (Reference values: 70 to 100 %); APTT 1.26 (Reference values: <1.20); albumin 41 g/l (Reference values: 32 to 46 g/l) and alpha fetoprotein 2.98 ng/ml (Reference values: <10 ng/ml). Hepatic magnetic resonance imaging(MRI) showed a large hepatic mass occupying segments 6 and 7, measuring 12.2 × 11.5 × 9.62 cm of heterogeneous tissue signal and a central liquid zone or necrosis, and suspicious intense signal in diffusion with ADC restriction for the most part. This large hepatic mass virtually contacts the right upper renal pole without infiltrating it, without infiltrating neighboring peri-hepatic fat, and without hepatic vascular or hilar extension. (Fig. 1). Extension workup showed no secondary sites.

The patient's medical file was discussed in a multidisciplinary meeting (MDT), and the decision was taken to perform an open right hepatectomy since future liver volume was sufficient. She was taken to the operating room on July 10, 2023. Intraoperative exploration revealed that the tumor measuring 25 cm of major axis occupying the entire right liver, compressing the inferior vena cava, no peritoneal carcinosis and no additional lesion in the remaining liver upon intraoperative ultrasound. A right hepatectomy was performed. The operation lasted 320 min and caused an estimated blood loss of 500 ml. The postoperative course was uneventful, and the patient was discharged on postoperative day 7.

**Fig. 2:**
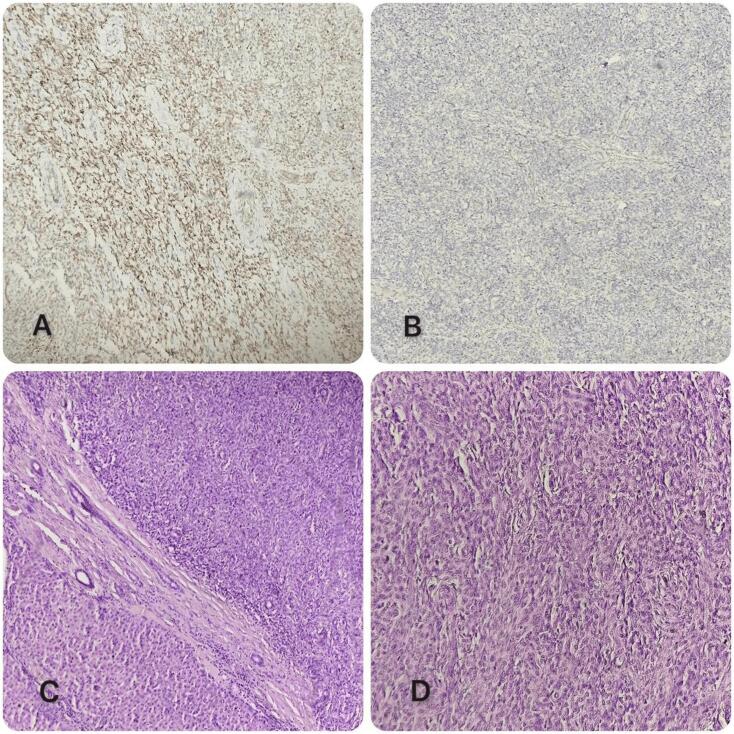
Histological images. A: Positive labeling of tumor cells with anti-TLE1 antibody. B: Absence of anti-CD117 antibody labelling of tumor cells. C: Morphological aspect showing on HE staining hepatic parenchyma infiltrated by proliferation arranged in diffuse sheets. D: Morphological appearance showing on HE staining a tumor proliferation of diffuse cell layers. Cells are medium-sized and fairly monomorphic.

Anatomopathological analysis of the surgical specimen favored an undifferentiated tumor process, requiring an immunohistochemical phenotyping study which showed positive staining for EMA, TLE-1, CD99 and Bcl-2. While CD117, DOG1, S100, Desmin and CD34 were negative. These findings led to the diagnosis of a monophasic synovial sarcoma, grade II (Intermediate) of FNCLCC (Fédération Nationale des Centres de Lutte Contre le Cancer) (Fig. 2). Surgical margins were clear at 0.5 cm.

**Fig. 3:**
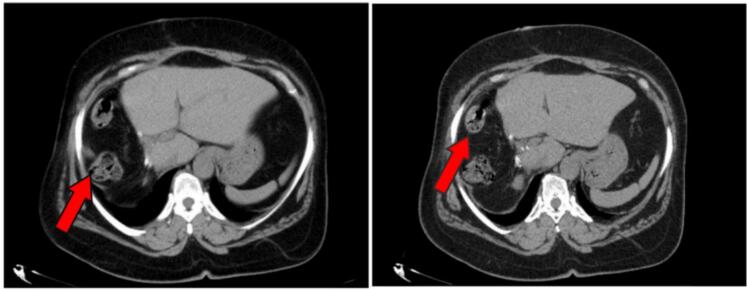
CT images of control objectifying the absence of local recurrence, presence of some a few bowel in the hepatectomy compartment indicated by the red arrow. (For interpretation of the references to colour in this figure legend, the reader is referred to the web version of this article.)

These histological findings were presented in MDT, and the decision was medical surveillance through regular follow-up appointments, including MRI and CT imaging. The patient was clinically healthy at the last check-up, with no complaints and a wholly healed surgical wound. The follow-up CT scan performed seven months after surgery was satisfactory, showing satisfactory left hepatic hypertrophy and no signs of suspicious or secondary localization (Fig. 3).

## 3 Discussion

### 3.1 Epidemiology/etiopathogenic

Soft tissue sarcomas are rare, accounting for 1 % of adult malignancies [15]. Synovial sarcoma is adults' third most common soft-tissue sarcoma, accounting for around 10 % [16]. Hepatic localization is extremely rare. In fact, we undertook searches of PubMed databases using the following keywords: "liver," "synovial sarcoma,"; "liver," "synovialosarcoma," and "liver," "synovial sarcomas." Only 13 cases were reported in the literature [[2], [3], [4], [5], [6], [7], [8], [9], [10], [11], [12], [13]]. Indeed, the origin of the cell that gives rise to this type of tumor is still unclear. Contrary to popular belief, "synovial" does not originate in synovial tissue. Most current studies suggest that it derives from primitive mesenchymal cells in mesenchymal tissue [17]. However, studies have shown that synovial sarcomas present a chromosomal translocation. It is thought to carry the t (X; 18) gene, resulting from the fusion of two genes, SS8 (in 18q11) and SSX (1, 2 or 4 in Xp11), forming the SS18-SSX gene fusion product [16]. There are three histological subtypes: the monophasic form observed in our patient, the biphasic form and the poorly differentiated form. The monophasic form comprises hypercellular arrays of small spindle cells with uniform, ovoid, vesicular nuclei with dispersed chromatin, discrete nuclei and very rare amphophilic cytoplasm. The biphasic form is made up of a mixture of epithelial components and spindle cells, in variable proportions. The poorly differentiated form generally comprises sheets of small, rounded cells, with hyperchromatic nuclei and amphophilic cytoplasm, frequent mitotic activity and necrosis [18]. The monophasic type observed in our patient is the most frequent in the literature, with 6 cases of monophasic type, 5 cases of biphasic type, one case of poorly differentiated type and one unspecified case (Table 1). Although our patient was of advanced age (68), synovial sarcoma (SS) is prevalent mainly in older children and young adults [18]. The hepatic form is no exception: of the 13 cases reported, 10 were under 50 years of age. Age ranged from 11 to 71 years, with an average of 38.30 years (Table 1). In our case, the subject was female, but the male sex is the most represented in synovial sarcoma of the liver, with eight men out of the 13 cases, i.e. a rate of 61.53 % (table1).

**Table 1:** Summary cases of synovial sarcoma of the liver reported in literature.

Case/Ref	Year of publication	Age/Gender	Clinical manifestations	Part of liver/Maximum diameter of tumor (cm)	Immunohistochemical forms/Immunohistochemical features	Therapeutics	Evolution/Prognosis
1/[2]	2005	60/F	Right upper quadrant pain + intrahepatic bleeding	Right lobe/10	Monophasic synovial sarcoma/vimentin(+), BCL-2(+), AE1(-), AE3(-), CAM5.2(-), CK7(-), CK20(-), S-100(-), HMB-45(-), MART-1(-), CD34(-), CD99(-).	Surgery (right partial hepatectomy) + adjuvant chemotherapy	Death 3 months later. Generalized metastaticdisease
2/[3]	2006	18/F	Fatigue, nausea and vomiting	Left lobe/21	Monophasic synovial sarcoma/AE1(+), AE3(+), CD99(-), CD34(-), CD117(-).	Surgery	NR
3/[4]	2011	44/M	Acute abdomen and severe pain in right upper quadrant	Left lobe/14	Monophasic synovial sarcoma/vimentin(+), CD99(+), Bcl-2(+), EMA(+), CKWS(+), CD31(-), CD34(-), S-100(-), HMB45(-), NSE(-).	Surgery (resection of 2 segments of the left hepatic lobe); the patient refused chemotherapy	Recurrence 6 monthslater; then lumpectomy + chemotherapy; recurrence again 2 months later with extension to the retroperitoneum, death 13 months later.
4/[5]	2013	13/M	Right upper quadrant pain	Right lobe/8.6	Monophasic synovial sarcoma/vimentin(+), BCL-2(+), TLE-1(+), CK7(+), Dog1(-), CD117(-), CD34(-), S-100(-), CD99(-), HMB-45(-).	Surgery (right hepatectomy) + 4 courses of chemotherapy	Local recurrence 11 months later
5/[6]	2015	21/F	Abdominal pain, emesis, diarrhea. et al	Caudate lobe/19	Monophasic synovial sarcoma/CK19(+), CDX2(+), SALL4(+), CK7(+), CD10(+), DPC(+), CD34(-), SMA(-), S-100(-), CD117(-).	Surgery	No relapse during 3 months follow-up
6/[7]	2015	49/M	Acute abdominal pain	Right lobe/12	Biphasic synovial sarcoma/vimentin(+), CD99(+), Bcl-2(+), EMA(+), CK8(+), CD34(-), EMA(-), S-100(-), SMA(-).	Surgery	No recurrence 3 months after surgery
7/[8]	2016	42/M	Flatulence, abdominal pain. et al	Right lobe/11.8	Monophasic synovial sarcoma/VIM(+), CD99(+), Bcl-2(+), PCK (+), CD34(-), EMA(-), S-100(-), CD117(-), DOG1(-), DES(-), Melan-A(-), HMB45(-), SMA(-), GFAP(-).	Surgery	Relapse 3 years later
8/[9]	2018	24/M	fever	right lobe/NR	Hypo-differentiated/vimentin (+), CD99 (+), AE1/AE3 (focal+), EMA9 (focal+), CD117 (-), CD31 (-), CLA (-), CgA (-). CD34 (-), DES (-), Syn (-), S-100 (-), Actin(-)	Surgery (right hepatectomy) + 5 courses of chemotherapy	Recurrence after 18 months of surgery
9/[10]	2022	11/F	intermittent nausea, vomiting and abdominal pain	right lobe/13.6	biphasic synovial sarcoma/CK pan (+), CK7 (+), vimentin (+), CD99 (+), TLE (+), CD117 (+), myogenin (-), CK20 (-), PAX-8 (-), WT-1 (-), GPC-3 (-), SFI (-), SMA (-), S-100 (-), CD34 (-), DES9 (-). Ki-67 (20 %+)	Surgery (partial right hepatectomy); the patient's family refused all adjuvant treatment	No recurrence until submission of article
10/[11]	2022	48/M	Right upper quadrant pain	Right lobe/11.6	Biphasic synovial sarcoma/vimentin (+), CD56 (+), TLE1 (+), FL.i1 (+), H3K27Me3 (+), INI1 (+), low relative molecular weight cytokeratin (+), CK18 (poly-like composnent +), CD99 (-), NY-ESO-1 (-), CD34 (-), S-100 (-), SOX10 (-), SMA (-), STAT6 (-), HepPar-1(-). Arginase-1 (-). ERG (-), DES (-), CgA (-)	Surgery	No recurrence 8 months after surgery
11/[11]	2022	52/M		Right lobe/8	Biphasic synovial sarcoma/vimentin (+), CD56 (+), TLE1 (+), FL.i1 (+), H3K27Me3 (+), INI1 (+), low relative molecular weight cytokeratin (+), CK18 (poly-like component +), CD99 (-), NY-ESO-1 (-), CD34 (-), S-100 (-), SOX10 (-), SMA (-), STAT6 (-), HepPar-1(-). Arginase-1 (-). ERG (-), DES (-), CgA (-)	Surgery + adjuvant chemotherapy	No recurrence 3 months after surgery
12/[12]	2022	45/M	Headaches, back pain	Right lobe/NA	Biphasic synovial sarcoma/vimentin (+), CKIS (+), Bd-2 (+), TFE3 (+), CD99 (+)	NR (patient transfer)	NR
13/[13]	2024	71/F	Abdominal pain	Right lobe and caudate lobe/11.5	NR/vimentin (+), EMA (+), H3K27Me3 (+), BCL-2 (+) et TLE1 (+), CK-pan (-), S-100 (-)	The patient's family abandoned the treatment	NR, loss of life

Legende: Cm: centimeter; F: female; M: male; NA: not available; NR: not reported; Ref: reference.

### 3.2 Clinical/paraclinical study

Our patient's history revealed no risk factors for synnovial sarcoma. However, risk factors common to soft tissue sarcomas are described. These include genetic predispositions such as Li-Fraumeni syndrome, familial adenomatous polyposis (FAP), Gardner syndrome, retinoblastoma syndrome, neurofibromatosis type I (Von Recklinghausen disease), neurofibromatosis type II, basal cell neuromatosis, Bourneville tubular sclerosis and Werner syndrome. There are also ionizing radiation, chemical agents and a family history of cancer [1]. Clinical signs of synovial sarcoma depend on the organ affected. General signs include unexplained weight loss, anorexia, asthenia and other functional signs (nausea, vomiting, etc.). In the case of synovial sarcoma of the liver, symptoms may include pain in the right hypochondrium (as in our patient's case), epigastric pain, pruritus, jaundice or the sensation of a hypogastric mass. Symptoms are often silent until the mass enlarges and compresses the surrounding organs [1]. According to case reports, clinical signs are dominated by abdominal pain, most often localized in the right hyponchondrium. A few cases of asthenia, nausea, vomiting and fever have been reported (Table 1). Most lesions are located in the right lobe, as in our patient, in 9 cases out of 13, i.e. almost 70 % of cases, followed by the left lobe with 2 cases or 15.4 %, one case in the caudate lobe and one case of simultaneous involvement of the right and caudal lobes (Table 1). Liver damage can give rise to biological signs such as anemia, thrombocytopenia, liver enzyme disorders or increased alpha-fetoprotein levels, which may suggest liver damage. None of these signs were observed in our patient, and all values were normal. The radiological signs of synovial sarcoma are not specific, but abdominal ultrasound, abdominal CT or PET scan can contribute to the diagnosis. Hepatic MRI remains the examination of choice for characterizing any suspicious lesion of the liver in general, and hepatic synovial sarcoma in particular. It identifies a triple-signal appearance with areas of low signal (calcifications and collagenous fibrosis), intermediate signal (cellular) and high signal (hemorrhagic and necrotic) in T2 [19]. To retain the diagnosis, specific differential diagnoses such as hepatocellular carcinoma must be ruled out by looking for a history of hepatic virosis and a significant elevation of alpha-proteins. Our patient had neither a history of hepatic viral infections nor elevated alpha-to-proteins.

### 3.3 Treatment/prognosis

Surgical excision is the standard treatment for localized sarcomas. The aim is to achieve complete R0 resection. Chemotherapy and radiotherapy are considered for advanced stages, but may be combined with surgery in cases of incomplete resection (R1 or R2) [1]. Due to its rarity, there is no standard protocol for synovial sarcoma of the liver. Radical resection is recommended for localized forms without metastases, or first-line adjuvant chemotherapy (adriamycin combined with cyclophosphamide) [13]. For unresectable and metastatic forms, radiotherapy is recommended according to the protocol for advanced forms of synovial sarcoma of the liver [20]. But targeted therapy in the treatment of synovial sarcomas has advanced considerably in recent decades. In addition to pazopanib, a receptor tyrosine kinase (RTK) inhibitor, which has so far proved its worth, other targeted therapies are showing promise [21]. None of the listed cases of synovial sarcoma of the liver benefited from targeted therapy. Of the 13 cases identified, 11 underwent surgical resection, 5 of which were followed by adjuvant chemotherapy. The remaining 2 cases were diagnosed as metastases, one to bone, the other to liver and lung [13]. As there is no standard protocol, we recommend that all cases, whatever their stage, be discussed in a multidisciplinary meeting (MDT), as in our patient's case.

According to the literature, the prognosis for liver disease is poor. Affected patients are likely to present with recurrence or early metastasis, the preferred sites of metastasis being liver, lung and bone. Of the 5 cases who underwent surgical excision followed by adjuvant chemotherapy, only one was free of recurrence for more than a year tab. The decision to monitor our patient without adjuvant chemotherapy was motivated by the success of a complete surgical excision (R0). This decision was taken in a multidisciplinary meeting. So far, the patient is doing better, as shown by the follow-up CT scan (Fig. 3). Long-term follow-up of the various cases reported in the literature has not been reported.

## 4 Conclusion

Synovial Sarcoma of the liver is a rare soft-tissue sarcoma with non-specific clinical and radiological manifestations. Diagnosis is based on anatomopathological analysis followed by immunohistochemical complementation. The therapeutic protocol is not standardized, but surgical excision is the reference treatment for localized forms. The prognosis is poor, with a recurrence rate of 80 % for forms that have benefited from surgical excision combined with adjuvant chemotherapy. Hence the need for regular follow-up. However, hope is still possible, as significant advances have been made in targeted therapies and immunotherapy.

## Author contribution

All authors (Moctar Noufou Fodiya, Oumayma Lahnaoui, Mouna Khmou, Mohammed Anass Majbar, Amine Souadka and Amine Benkabbou) contributed to the design, analysis and interpretation of the data, as well as the writing of this study.

## Consent

Written informed consent was obtained from the patient for publication and any accompanying images. A copy of the written consent is available for review by the Editor-in-Chief of this journal on request.

## Ethical approval

This study is exempt from ethical approval in our institute because it is a case report.

## Guarantor

All authors are guarantors, accept full responsibility for this study and have approved the publication of this study.

Principal guarantor: Moctar Noufou Fodiya.

## Research registration number

None.

## Funding

All authors report that they have not received any financial support from any organization or individual for the submitted work.

## Conflict of interest statement

None of the authors have any conflicts of interest to declare.
